# SARS-CoV-2 Infection of Rhesus Macaques Treated Early with Human COVID-19 Convalescent Plasma

**DOI:** 10.1128/Spectrum.01397-21

**Published:** 2021-11-24

**Authors:** Jesse D. Deere, Timothy D. Carroll, Joseph Dutra, Linda Fritts, Rebecca Lee Sammak, JoAnn L. Yee, Katherine J. Olstad, J. Rachel Reader, Amy Kistler, Jack Kamm, Clara Di Germanio, Yashavanth Shaan Lakshmanappa, Sonny R. Elizaldi, Jamin W. Roh, Graham Simmons, Jennifer Watanabe, Rachel E. Pollard, Jodie Usachenko, Ramya Immareddy, Brian A. Schmidt, Shelby L. O’Connor, Joseph DeRisi, Michael P. Busch, Smita S. Iyer, Koen K. A. Van Rompay, Dennis J. Hartigan-O’Connor, Christopher J. Miller

**Affiliations:** a Department of Medical Microbiology and Immunology, School of Medicine, University of California Davis, Davis, California, USA; b Center for Immunology and Infectious Diseases, University of California Davis, Davis, California, USA; c California National Primate Research Center, University of California Davis, Davis, California, USA; d Chan Zuckerberg Biohub, San Francisco, California, USA; e Vitalant Research Institute, San Francisco, California, USA; f Department of Laboratory Medicine, University of California San Francisco, San Francisco, California, USA; g Department of Pathology and Laboratory Medicine, University of Wisconsin-Madison, Madison, Wisconsin, USA; h Wisconsin National Primate Research Center, University of Wisconsin-Madison, Madison, Wisconsin, USA; i Department of Biochemistry and Biophysics, University of California San Francisco, San Francisco, California, USA; j Department of Pathology, Microbiology, and Immunology, School of Veterinary Medicine, University of California Davis, Davis, California, USA; k Department of Internal Medicine, Division of Infectious Diseases, School of Medicine, University of California Davis, Davis, California, USA; Fundacio irsiCaixa

**Keywords:** SARS-CoV-2, COVID-19, convalescent plasma, passive immunization, nonhuman primate, animal models of infectious diseases, microbial pathogenesis, virology

## Abstract

Human clinical studies investigating use of convalescent plasma (CP) for treatment of coronavirus disease 2019 (COVID-19) have produced conflicting results. Outcomes in these studies may vary at least partly due to different timing of CP administration relative to symptom onset. The mechanisms of action of CP include neutralizing antibodies but may extend beyond virus neutralization to include normalization of blood clotting and dampening of inflammation. Unresolved questions include the minimum therapeutic titer in the CP units or CP recipient as well as the optimal timing of administration. Here, we show that treatment of macaques with CP within 24 h of infection does not reduce viral shedding in nasal or lung secretions compared to controls and does not detectably improve any clinical endpoint. We also demonstrate that CP administration does not impact viral sequence diversity *in vivo*, although the selection of a viral sequence variant in both macaques receiving normal human plasma was suggestive of immune pressure. Our results suggest that CP, administered to medium titers, has limited efficacy, even when given very early after infection. Our findings also contribute information important for the continued development of the nonhuman primate model of COVID-19. These results should inform interpretation of clinical studies of CP in addition to providing insights useful for developing other passive immunotherapies and vaccine strategies.

**IMPORTANCE** Antiviral treatment options for severe acute respiratory syndrome coronavirus 2 (SARS-CoV-2) remain very limited. One treatment that was explored beginning early in the pandemic (and that is likely to be tested early in future pandemics) is plasma collected from people who have recovered from coronavirus disease 2019 (COVID-19), known as convalescent plasma (CP). We tested if CP reduces viral shedding or disease in a nonhuman primate model. Our results demonstrate that administration of CP 1 day after SARS-CoV-2 infection had no significant impact on viral loads, clinical disease, or sequence diversity, although treatment with normal human plasma resulted in selection of a specific viral variant. Our results demonstrate that passive immunization with CP, even during early infection, provided no significant benefit in a nonhuman primate model of SARS-CoV-2 infection.

## INTRODUCTION

Infection with severe acute respiratory syndrome coronavirus 2 (SARS-CoV-2) can be asymptomatic or can lead to potentially life-threatening coronavirus disease 2019 (COVID-19) in a minority of patients, particularly in aged people and those with comorbidities (1, 2). SARS-CoV-2 infections typically clear rapidly, with little detectable nucleic acid in the respiratory tract 9 days after symptom onset (3). Nonetheless, there is significant variation in the duration of virus shedding, with severe infection associated with prolonged shedding (4). The mechanisms driving variable pathogenesis are incompletely understood, although patients with severe disease are more likely to have polymorphisms in several genes impacting host antiviral defense or inflammation, such as oligoadenylate synthetase (*OAS*) and tyrosine kinase 2 (*TYK2*) (5). Detection of virus in the blood (viremia) is also associated with increased disease severity and mortality (6).

After virus clearance, a subset of individuals require hospitalization due to aberrant immune responses and inflammation that damage the lungs. Antiviral therapies that may be useful in the early phase of infection have little effect after hospitalization, possibly due to the temporal separation between maximal viral replication and inflammatory consequences (7–9). Conversely, anti-inflammatory/immunosuppressive therapies that allow uncontrolled virus replication if given soon after infection are successfully used to treat late-stage severe disease caused by inflammation (10). The finding that administration of dexamethasone increases mortality if given early following infection but significantly reduces the rate of mortality in patients on ventilators (10) corroborates the important contribution of postviral immunopathology to severe disease.

Early in the pandemic, there were few antiviral treatment options, and care was primarily supportive. Convalescent plasma (CP) (i.e., plasma from recovered COVID-19 patients) was therefore investigated as a potential treatment. CP was used with some success in treating patients with severe disease (11), and preliminary clinical results suggested that CP might reduce viremia (12). In August 2020, based on data from the Mayo Clinic-led expanded access program (EAP), the FDA provided an emergency use authorization for CP in treating COVID-19 (13). Although three randomized controlled clinical trials of CP demonstrated no significant benefit in patients with severe disease (7–9), CP-treated patients in a fourth study had increased rates of negative PCR tests, suggesting a direct antiviral effect (14). Further, it was recently shown that administration of high-titer CP against SARS-CoV-2 to mildly ill older adults within 72 h of symptom onset reduces the progression of COVID-19 (15). However, a recent meta-analysis of published studies examining the efficacy of CP showed no benefit, resulting in closure of the National Heart, Lung, and Blood Institute Clinical Trial of COVID-19 Convalescent Plasma in Outpatients (NHLBI C3PO) in emergency departments for futility (16). Results of the Randomized Evaluation of COVID-19 Therapy (RECOVERY) trial using high-titer CP were also recently published, demonstrating no apparent efficacy (17). One limitation of these clinical findings, in terms of the potential use of CP as an antiviral therapy, is timing of treatment initiation based on symptom onset.

We hypothesized that early administration of CP, within 24 h of SARS-CoV-2 infection, would reduce viral replication and lead to more rapid viral clearance. To test this hypothesis, we used a nonhuman primate (NHP) model of SARS-CoV-2 infection. Rhesus macaques are susceptible to the virus and recapitulate features of mild to moderate disease in humans (18, 19). The macaque model has been widely used to test candidate SARS-CoV-2 vaccines for immunogenicity and efficacy (20–22). The primary goal of this study was to determine if human CP, administered early in infection, reduces viral shedding in the rhesus macaque model.

## RESULTS

### Challenge of rhesus macaques with SARS-CoV-2 results in robust viral replication, mild clinical signs, and mild pathological lung findings.

**FIG 1:**
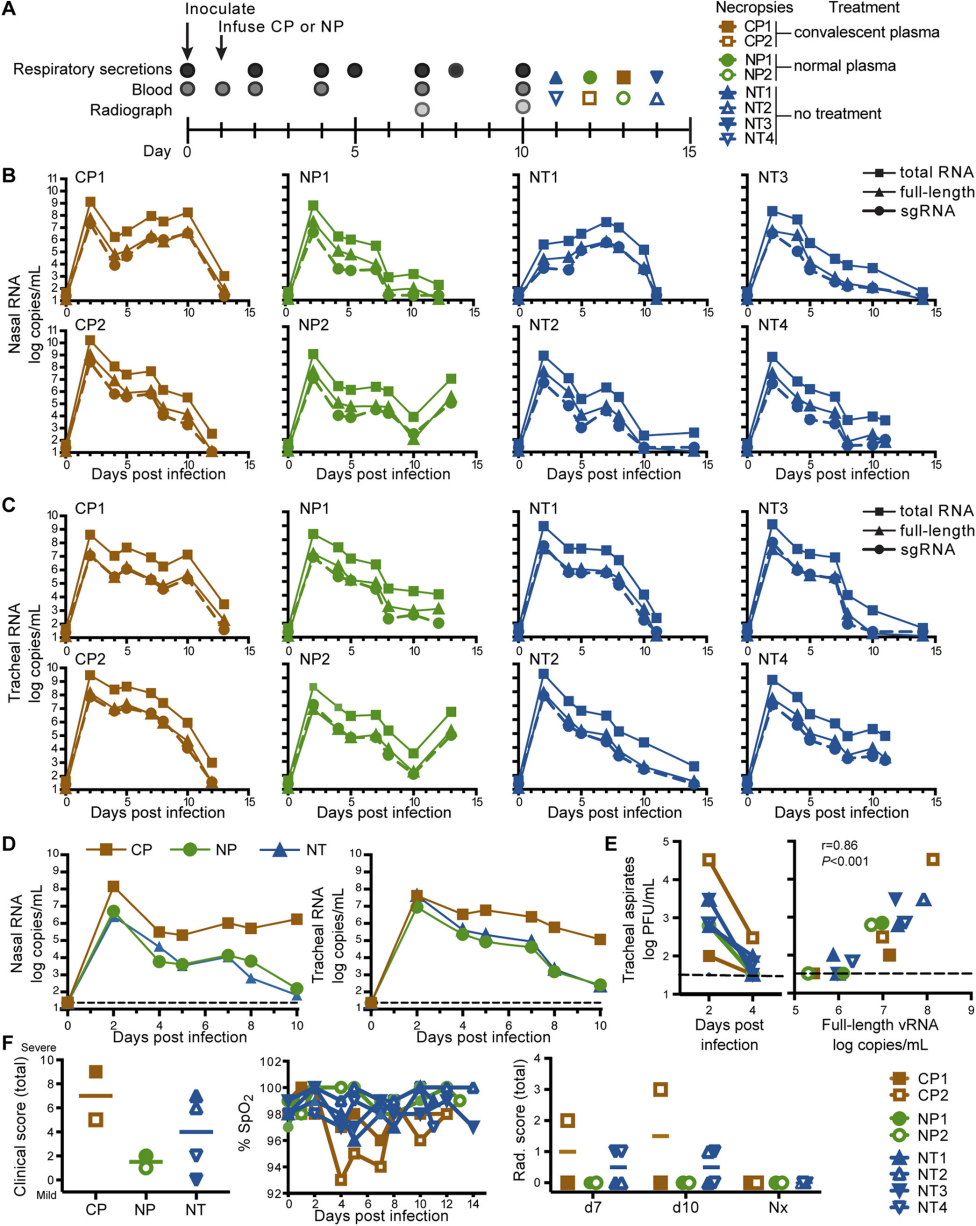
Impact of CP treatment on viral RNA and clinical outcomes. (A) Study design. (B, C) Quantitative PCR analysis of total vRNA, full-length vRNA, and subgenomic RNA (sgRNA) in nasal lavages (B) and tracheal aspirates (C) from individual animals over time. Graphs show mean of four independent qPCRs for each RNA target. (D) Median sgRNA levels in nasal lavages and tracheal aspirates of each experimental group over time. The dashed line indicates limit of detection. (E) Levels of infectious virus in tracheal aspirates as determined by plaque assay. Right graph shows the correlation between full-length vRNA (gRNA) detected by qPCR and viral plaque counts in these samples. (Spearman correlation *r* = 0.86, *P* ≤ 0.001). (F) Clinical outcomes, including total clinical scores, blood oxygen content (% oxygen saturation [SpO_2_]), and total radiographic (Rad.) scores. Nx indicates those samples collected at the time of necropsy. Horizontal lines on scatterplots show medians; *n *=* *2 (CP treatment), *n *=* *2 (NP treatment), and *n *=* *4 (not treated [NT]).

As previously described (23), eight macaques were inoculated with 2 × 10^6^ PFU of SARS-CoV-2 via a combination of intratracheal (1 ml), nasal (0.5 ml per nostril), and intraocular (one drop per eye) routes. Twenty-four hours after infection, two animals were intravenously infused with human COVID-19 convalescent plasma (CP; 4 ml/kg body weight), two animals were intravenously infused with normal human plasma (NP; 4 ml/kg body weight), and the four remaining animals received no plasma treatment (NT; Fig. 1A and Table 1). To increase the applicability of the findings to diverse CP donors, a CP pool was created by combining equal parts of plasma aliquots from four individual CP donors. Anti-SARS-CoV-2 antibodies in each donor plasma aliquot, assessed using a VITROS immunoassay, had titers of 8.18, 18.3, 35.6, and 60.7 signal/cutoff (S/C). The combination resulted in a pool that had a 50% neutralization titer (NT_50_) of 1,149 in a reporter virus assay (23). The animals were necropsied 11 to 14 days after infection.

**TABLE 1:** Description of study animal characteristics

Treatment	Code	Age at enrollment (mo)	Sex	Wt (kg)
Convalescent plasma	CP1	72	M	8.83
Convalescent plasma	CP2	72	M	8.43
Normal plasma	NP1	72	M	8.95
Normal plasma	NP2	71	M	9.74
No treatment	NT1	60	F	7.01
No treatment	NT2	60	F	5.47
No treatment	NT3	60	M	10.72
No treatment	NT4	72	F	6

Nasal lavages and tracheal aspirates were collected regularly and assessed by quantitative real-time PCR (qPCR) for full-length genomic viral RNA (gRNA; open reading frame 1a [ORF1a] target amplicon), subgenomic RNA (sgRNA; leader/Nucleocapsid [N] target) and total viral RNA (vRNA; N target). Peak viral loads in both nasal lavages (Fig. 1B) and tracheal aspirates (Fig. 1C) were reached in most animals at 2 days after infection before subsequently declining. Treatment with CP did not blunt initial viral replication nor did it accelerate clearance of gRNA, sgRNA, or total vRNA in either nasal lavages or tracheal aspirates (Fig. 1D; Fig. S1A and B in the supplemental material). An area under the curve analysis demonstrated that CP treatment did not reduce total viral burden in respiratory secretions from either the nose or trachea (Fig. S1C).

We confirmed that detection of SARS-CoV-2 gRNA and sgRNA in tracheal aspirates reflected the presence of replication-competent SARS-CoV-2. Infectious virus was detected in plaque assays using samples collected 2 and 4 days after infection; unexpectedly, the highest virus level was in CP-treated animal CP2 (Fig. 1E, open brown squares). Similar to results obtained using human samples (3), live virus was typically recoverable from samples containing >10^6^ genome copies/ml (Fig. 1E). The number of genome copies detected by PCR and replication-competent particles detected by plaque assay were correlated (Spearman correlation *r* = 0.86, *P < *0.001), and we detected one infectious virion per ∼10^6^ copies of gRNA (full length).

Treatment with CP did not appear to have a significant impact on the clinically mild disease course; treated and untreated animals had infrequent coughing, sneezing, and minimal nasal discharge (Fig. 1F). One CP-treated animal (CP2) had decreased oxygen saturation and the highest radiographic scores postinoculation of all the animals (Fig. 1F). The radiographic changes in CP2 on day seven were consistent with mild interstitial pneumonia that resolved and was inapparent by necropsy. This animal also had elevated plasma levels of two inflammatory markers, interleukin-6 (IL-6) and C-reactive protein (CRP), 2 days after infection (Fig. S2). A naive control animal (NT2) developed dermatitis on day seven, with erythema and scale on the ventrum and distal extremities, which worsened on the eighth day and then decreased through the experimental endpoint on day 14. Body temperatures and weights remained stable for all animals throughout the study.

### Transfusion of macaques with human CP achieves detectable binding but not neutralizing antibody titers in recipients.

**FIG 2:**
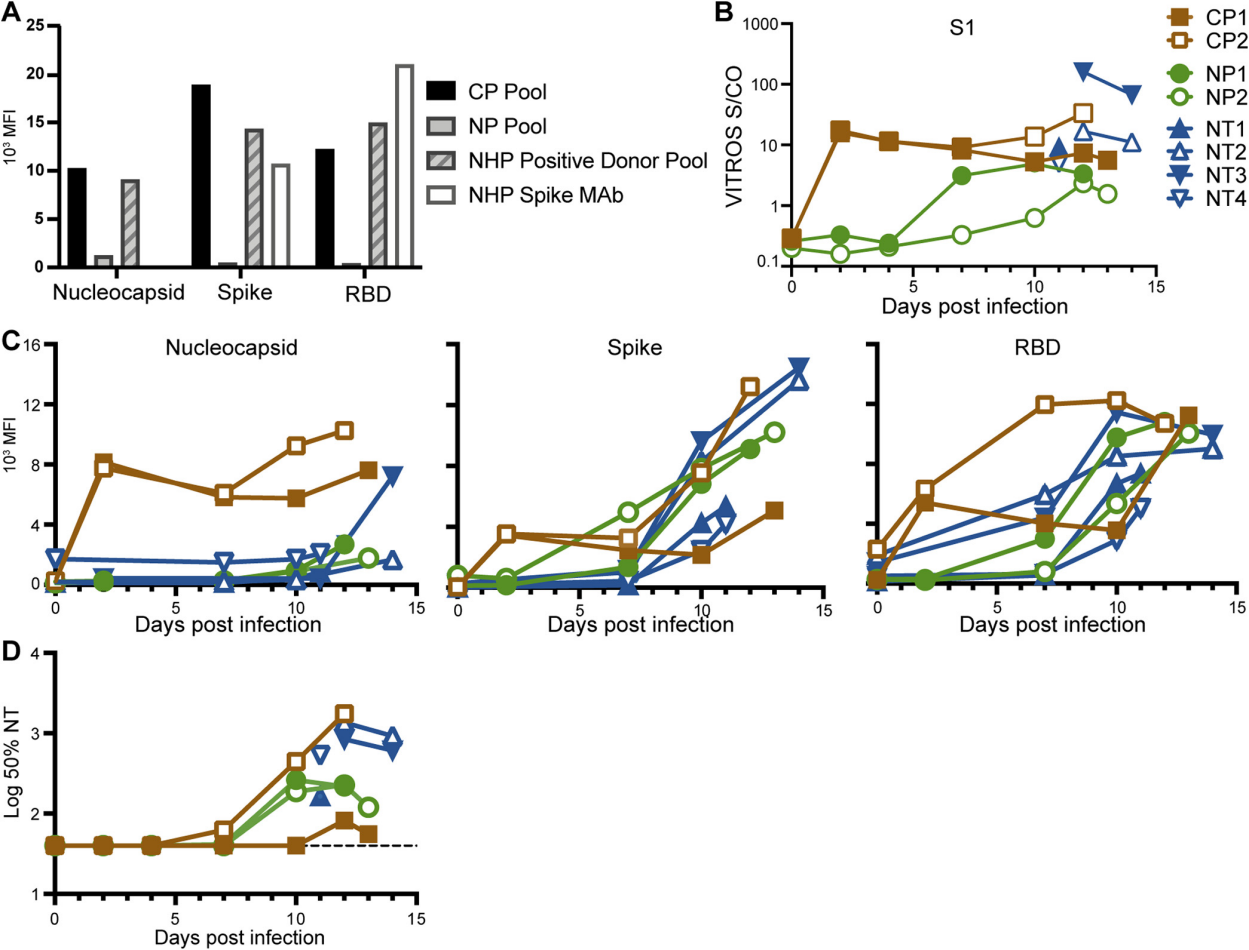
Antibody levels to SARS-CoV-2 in CP and plasma of animals. (A) Binding antibody levels to the viral nucleocapsid, spike, and RBD antigens in the CP pool and NP pool used for transfusion, a rhesus macaque anti-SARS-CoV-2 antibody positive plasma pool positive control, and an NHP monoclonal antibody to SARS-CoV-2 spike (NHP spike MAb). (B) Total S1 antibody levels in the plasma of each animal over the course of the study using an antigen sandwich assay. (C) Antibody levels to nucleocapsid, spike, and RBD in the plasma of each animal over the course of the study using a multiplex assay. (D) Pseudovirus 50% neutralization titer (50% NT) levels in the plasma of each animal over the course of the study.

SARS-CoV-2-specific binding and neutralizing antibody responses were assessed in the donor plasma pools and longitudinally in plasma samples collected throughout the study. Both donor pools (CP and NP) were assessed for binding antibody responses to SARS-CoV-2 nucleocapsid, spike (S), and receptor-binding domain (RBD) using a Luminex assay (Fig. 2A). The CP donor pool antibodies bound all three antigens tested, while the NP donor pool antibodies did not bind to any of them. A positive-control nonhuman primate donor plasma pool acquired from BEI Resources reacted with all three antigens, and a nonhuman primate monoclonal anti-spike antibody from the Nonhuman Primate Reagent Source bound spike and RBD but not nucleocapsid.

Total antibody responses to the spike protein S1 subunit in longitudinal serum samples from the study animals were assessed using an emergency use authorization (EUA) antigen sandwich format S1 total Ig assay from Ortho Diagnostics (Fig. 2B). S1 total Ig titers in sera collected 1 day after CP administration were 15.9 (CP1) and 17.5 (CP2) S/C, demonstrating that sufficient CP was delivered to achieve medium antibody titers in the treated macaques (13). Binding antibody responses were also assessed using the Luminex multiplex antigen assay (Fig. 2C). The CP-treated animals had detectable binding antibodies 1 day after CP administration to all three of the SARS CoV-2 antigens tested in the Luminex assay, indicating successful passive transfer of antibodies to the animals. These binding antibodies remained detectable throughout the study. The earliest detection of binding antibodies in the other control animals, reflecting *de novo* synthesis, was at 7 days postinfection. All of the animals had antibody responses to spike and RBD by day 10 postinfection. Of note, only modest responses to nucleocapsid were detected in most animals. The exception was animal NT1; this animal mounted strong responses to all viral antigens despite similar vRNA levels in secretion samples as the other animals.

Pseudovirus neutralizing antibody responses were also assessed in longitudinal plasma samples from the study animals (Fig. 2D). In contrast to readily detectable binding antibodies following CP administration (Fig. 2C), no neutralizing activity was detected in CP-treated animals the day after treatment (the lowest dilution tested was 1:40) (23). All treated animals (CP and NP) generated neutralizing antibodies by the end of the study (Fig. 2D). Animal CP2, the animal that developed symptoms of mild pneumonia, had the highest neutralizing antibody responses, reaching an NT_50_ of 1,754 at the time of necropsy. Notably, animal NP2 had increasing levels of vRNA in both nasal lavages and tracheal aspirates but declining neutralizing antibody responses at the end of the study.

### CP treatment does not impact host cellular immunity.

**FIG 3:**
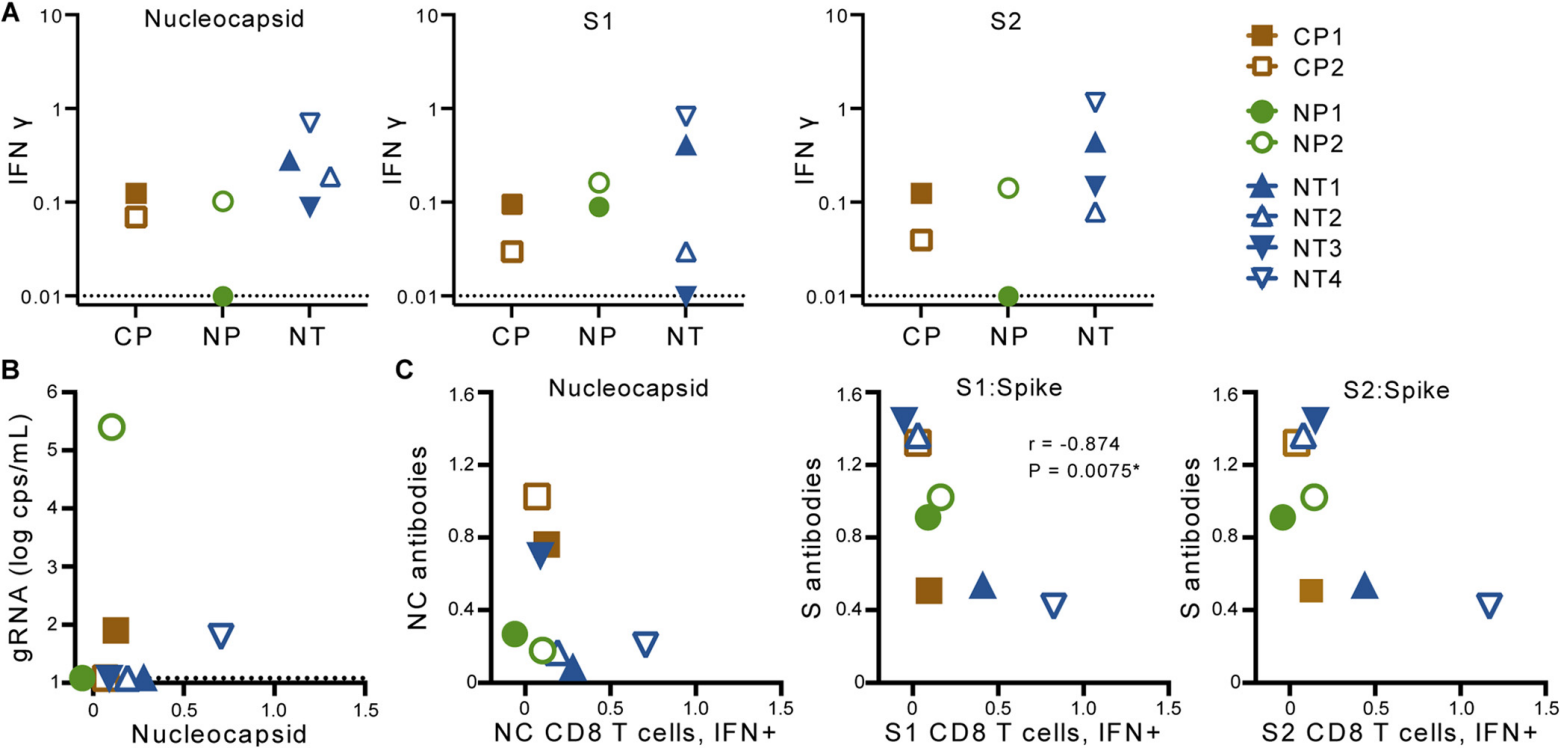
CD8 T-cell responses to SARS-CoV-2. (A) SARS-CoV-2-specific CD8 T cell responses in blood collected at necropsy. (B) Relationship between full-length vRNA (gRNA) copies (cps) in nasal lavages and nucleocapsid (NC)-specific CD8 T-cell responses at necropsy. (C) Relationship between antigen-specific antibody responses and corresponding CD8 T-cell responses assessed at necropsy. Results of a Spearman correlation are shown; IFN-γ, interferon-γ.

Despite failure to detect a virologic difference between groups, we considered the possibility that reduced viral replication due to CP treatment might be reflected in lower T-cell responses. In fact, low cellular responses were seen across all groups at these early time points (11 to 14 days postinfection) (Fig. 3A). Two animals with superior responses, NT1 and NT4, did not derive any apparent virologic benefit, with NT1 manifesting a lower peak viral load before adaptive responses were detectable and NT4 clearing virus at a comparable pace to other animals with weaker T-cell responses (Fig. 1B and C). Indeed, NT4 was among only three animals to have remaining detectable full-length genomes in the nares and trachea at necropsy (Fig. 3B).

CD8 T-cell and antibody responses against S1 were inversely correlated (Fig. 3C), suggesting the possibility that CD8^+^ cytotoxic T-cell responses can reduce the availability of antigen to drive plasmablast differentiation. We observed no correlation between the CD8^+^ cytotoxic T-cell responses described here and the T follicular helper (Tfh) cell responses that were previously reported in these animals (23).

### CP did not exert selective pressure on replicating SARS-CoV-2.

**FIG 4:**
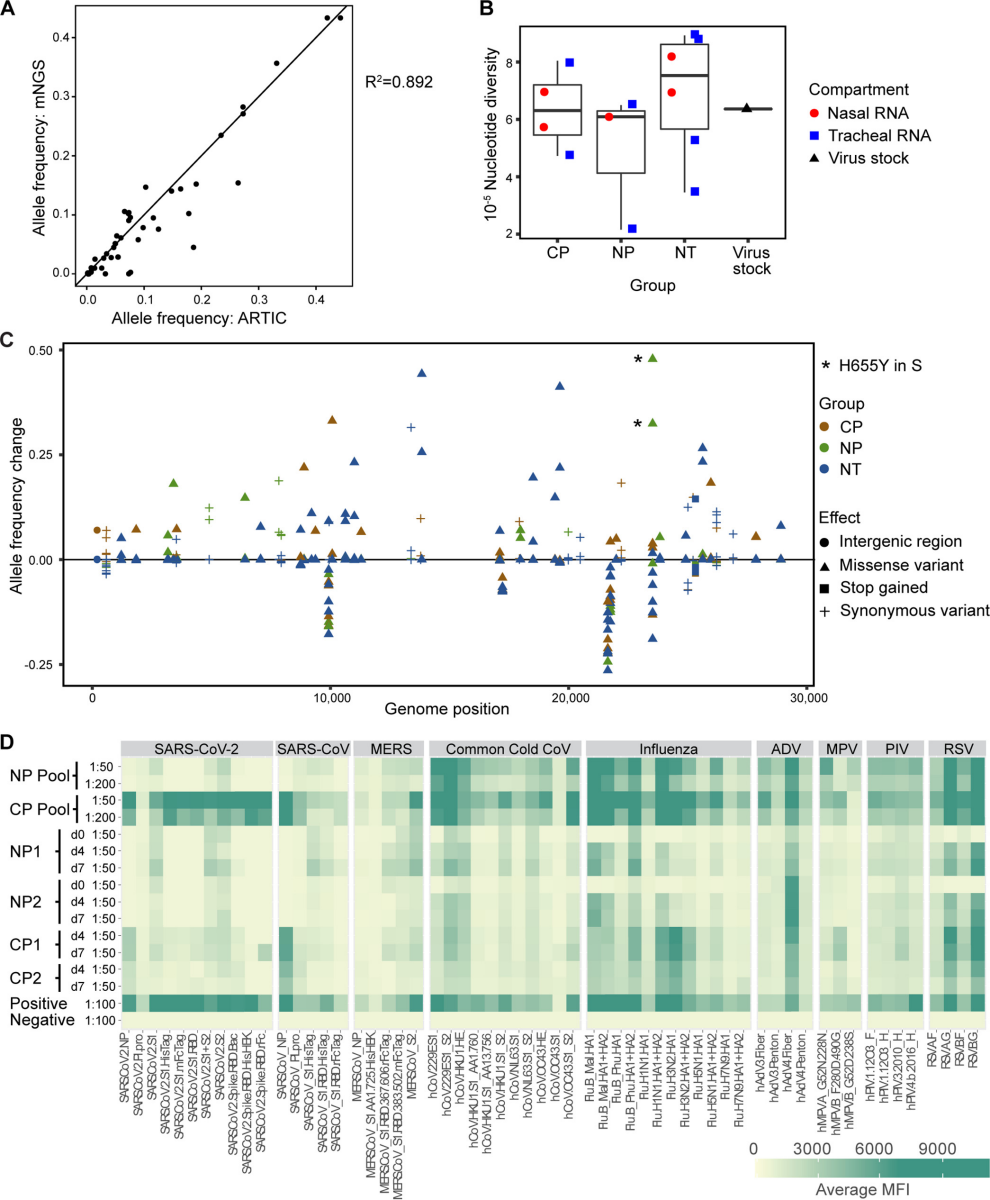
Impact of CP treatment on viral replication and evolution. (A) Comparison of allele frequencies determined using amplicon (ARTIC) and metagenomic sequencing (mNGS) methods. The square of the Pearson correlation coefficient values are shown. (B) Impact of treatment on viral sequence nucleotide diversity in nasal lavages and tracheal aspirates was assessed by ARTIC for comparison with the virus stock used for inoculation. A box plot of pairwise nucleotide diversity shows the median and the 1st and 3rd quartiles. (C) Impact of treatment on viral allele frequencies was assessed in comparison to the virus stock. Asterisks indicate expansion of a H655Y substitution in the viral spike protein. (D) Heat map showing antibody cross-reactivity to other related viruses in the human plasma pools and in plasma samples from study animals treated with NP and CP.

We assessed viral RNA in tracheal aspirates and nasal lavages for intrahost polymorphisms using amplicon (ARTIC v3) and metagenomic next-generation sequencing (mNGS). The amplicon-sequencing approach achieved >1,000× average read depth over the SARS-CoV-2 genome on all day seven samples and the inoculum (Fig. S3A). The necropsy samples had a wide range of average read depths due to variable amounts of remaining vRNA. Samples with lower viral loads (including necropsy samples) generated genome sequences with significantly lower read depths and correspondingly elevated error rates, as indicated by greater nucleotide diversity (Fig. S3B), potentially confounding estimates of intrahost variation. We thus removed all necropsy samples and three nasal lavages from subsequent analyses due to their lower viral loads (threshold cycle [*C_T_*] ≥ 26.5). After filtering, 13 genomes from the day seven time point (all eight tracheal aspirates and five nasal lavages) obtained via amplicon sequencing were assessed. mNGS recovered fewer genomes than ARTIC but was used as an orthogonal sequencing approach to validate the accuracy of intrahost variant allele frequencies; the two methods were concordant with *R*^2^ = 0.892 (Fig. 4A). Our analysis demonstrated no relationship between treatment and nucleotide diversity (Fig. 4B). In addition, no variants consistently increased in frequency in serial samples from CP-treated animals (Fig. S3C). Together, these data demonstrate that CP treatment had no discernible impact on viral evolution.

Unexpectedly, one polymorphism, H655Y in the spikes (S) gene, expanded in tracheal aspirates from both animals treated with NP, increasing from 25% in the inoculum to over 50% in both NP-treated animals (Fig. 4C, H655Y marked with asterisks). We speculated that cross-reactive antibodies to a seasonal coronavirus within the NP donor pool might have selected for expansion of this variant. The plasma donor pools and plasma samples from all treated animals were therefore assessed for antibody responses to SARS-CoV-2, SARS-CoV, Middle East respiratory syndrome coronavirus (MERS-CoV), various seasonal human coronaviruses (HCoV-229E, HCoV-NL63, HCoV-HKU1, and HCoV-OC43), influenza, and several other common cold viruses by using a coronavirus antigen microarray (CoVAM) (Fig. 4D). As expected, the NP pool had no or very low levels of reactivity to SARS-CoV-2, SARS-CoV, or MERS-CoV antigens. Both the NP and CP pools had readily detectable antibodies to S1 of the seasonal coronavirus HCoV-229E and lower but detectable responses to S1 of HCoV-HKU1 and HCoV-NL63. None of the plasma samples reacted with S1 of HCoV-OC43. Thus, the NP donor pool did not contain reactivity to a seasonal coronavirus S1 antigen that could explain selection of the H655Y substitution in SARS-CoV-2.

## DISCUSSION

We tested the impact of CP treatment 24 h postinfection in a nonhuman primate model of mild clinical disease. CP, administered early in infection, had no significant impact on viral loads in respiratory secretions, on clinical outcomes, or on the development of adaptive immune responses. In addition, histopathologic changes in the lungs (23) were similar in the animals with and without CP treatment. Although this study was limited by the number of animals that received CP treatment and the mild-to-moderate severity of disease in the model, several recent randomized clinical trials have demonstrated similar findings (7, 8, 14), including several studies that were stopped for futility in hospitalized patients with more severe disease (17, 24, 25).

There are conflicting reports on the efficacy of convalescent plasma treatment for SARS CoV-2 infection and its associated disease, COVID-19. Because there is no FDA-approved method for measuring neutralizing antibodies, some of the variation in CP efficacy among different studies could be due to variations in the volume and neutralizing antibody titer of the CP units used. In fact, a recent clinical trial reports that CP pools with higher neutralizing antibody titers are associated with increased clinical benefit (13). This finding is consistent with a recent report that African green monkeys (AGM) transfused 10 h postinfection with a pool of high-titer (50% plaque reduction/neutralization titer [PRNT_50_] of 2,048) convalescent AGM plasma had lower viral loads in the respiratory tract, reduced lung pathology and normalization of the prolonged coagulation times, elevated fibrinogen, thrombocytopenia, and hypercytokinemia seen in untreated animals (26). Our results with moderate-titer human CP in rhesus macaques are largely consistent with a report that low-titer AGM CP given 10 h postinfection had little effect on virus replication in AGMs (26).

Although there have been no documented instances of vaccine-associated enhanced disease (VAED) following coronavirus vaccinations in humans (27), the higher clinical scores and the high viral loads in respiratory secretions of our CP-treated macaques are striking. One conclusion that can be drawn is that low concentrations of antibodies, below a possible protective threshold (20), are unlikely to decrease virus replication. The apparent failure of CP to control viral replication in our study could be a result of the postinfusion dilution of the neutralizing antibodies in the CP below a protective threshold in recipients rather than antibody-dependent enhancement (ADE). The high clinical scores and viral loads observed in the CP-treated macaques could therefore be explained by normal variation in the macaque model. A determination is beyond the scope of this study due to limitations on study size and the current limitations in measuring VAED/ADE (28).

Recent studies have demonstrated that CP can drive viral diversity and variant selection (29–31). We considered the possibility that, while the administered CP was insufficient to have a measurable effect on virus replication, it may have been sufficient to exert immune pressure that would be revealed in metagenomic sequencing. Such mutations can occur in the case of many other RNA and DNA viruses, including hepatitis B virus (32), hepatitis C virus (33), human immunodeficiency virus (HIV) (34, 35), and many coronaviruses (36). No mutations appeared to have been selected by CP administration to macaques. However, we observed an increased frequency of a specific substitution (H655Y in S) in both animals that received normal human plasma. This residue is in the C-terminal domain (CTD) of the coronavirus spike protein, outside the RBD (37), and was previously reported as a likely cell culture adaptation (38). It is important to note that this substitution was present at a low frequency in the inocula. The increase in frequency observed in the NP-treated animals was suggestive of selective pressure, but we could find no evidence that this amino acid residue is the target of antibodies. We also considered the possibility that the normal human plasma pool that we used contained antibodies against the spike protein of a seasonal coronavirus that was cross-reactive with a neutralizing epitope on SARS-CoV-2. However, a survey of reactivity to seasonal coronaviruses in the plasma pools and plasma recipient monkeys revealed no unusual pattern in the two normal plasma recipients compared to other macaques in the study.

Comparison of our experience with CP administration to the effects of low-dose monoclonal antibodies should yield insight into the important question of whether nonneutralizing antibodies are detrimental in COVID-19. The CP we administered comprised both neutralizing antibodies (at a titer of 1:1,149 in a pseudovirus assay) and binding antibodies (at unknown concentration). The question of whether binding antibodies affect COVID-19 pathology remains important and may dictate the choice of immunogen in future vaccines. RBD-only immunogens present a restricted attack surface and a high chance of generating multiple neutralizing antibodies, while full-length spike immunogens elicit binding antibodies and neutralizing antibodies, and the specific ratio of these could accelerate disease. RBD antigens also elicit higher-affinity binding antibodies that correlate with virus neutralization (39).

Our results also add to the expanding collection of data on the course of SARS-CoV-2 infection in the rhesus macaque model. Seven of eight infected animals demonstrated a very mild disease course that is typical for this species, with rapid virologic control and minimal clinical symptoms. One of eight, however, experienced apparent virologic control in the first 10 days followed by a >3-log rebound in viral nucleic acid. The timing of this apparent loss of control is reminiscent of the timing in which human patients worsen when that occurs (40). The rhesus macaque model may in fact closely model the full spectrum of human disease with a high frequency of rapid control, with a small proportion of animals developing moderate disease. The relevance of the macaque model to the clinic might require studies using significantly more animals over a longer duration, both limitations of our study, to determine whether the few macaques that appear to lose virologic control progress to more severe disease.

In this study, human CP treatment provided no discernible benefit to SARS-CoV-2-infected rhesus macaques. In fact, both CP recipient animals had high viral loads, with one CP recipient having the highest vRNA levels and virus titers in respiratory secretions and highest clinical scores of any animal studied. Our findings demonstrate that postexposure therapy with medium-titer CP does not provide a virologic, clinical, or pathological benefit, even when administered within 24 h of infection.

## MATERIALS AND METHODS

### Experimental animals and samples.

This study was approved in advance by the University of California, Davis (UC Davis) IACUC and was performed at the California National Primate Research Center (CNPRC). Housing, medical care, and all procedures were performed in accordance with UC Davis IACUC-established policies. UC Davis has an Animal Welfare Assurance on file with the NIH Office of Laboratory Animal Welfare and is fully accredited by the Association for the Assessment and Accreditation of Laboratory Animal Care International. Eight rhesus macaques (Macaca mulatta) were selected from the conventional colony at CNPRC. All animals tested negative for HIV-2, simian immunodeficiency virus (SIV), simian T cell lymphotropic virus type 1 (STLV), type D retrovirus, and SARS-CoV-2 at the start of the study. Animals were housed in the animal biosafety level 3 (ABSL-3) laboratory at the CNPRC. Animals were administered 10 mg/kg body weight ketamine-HCl intramuscularly (i.m.) when necessary for immobilization. Analgesics were administered at the discretion of the CNPRC veterinary staff in an effort to minimize pain and discomfort. Nasal lavages and tracheal aspirates were collected and processed as previously described (41) in a biosafety level 3 (BSL-3) laboratory at UC Davis.

### Virus.

Stocks of SARS CoV-2 were prepared by propagation of a virus isolate acquired from a patient sample from the UC Davis Medical Center. Vero cells (ATCC, CCL-81) were used to isolate, expand, and assess the virus stocks. Passage 2 virus was assessed by sequence analysis (SARS-CoV-2/human/USA/CA-CZB-59 × 002/2020; GenBank accession number MT394528). The stock titer was determined by plaque assay as previously described (42), with the exception of a 0.9% low-melt agarose overlay, and was used to inoculate all eight rhesus macaques in this study. Replication-competent virus in nasal lavage and tracheal aspirate samples was also assessed using plaque assays.

### Convalescent plasma transfusions.

Pools of plasma from SARS-CoV-2 convalescent patients or healthy donors were provided by Vitalant Inc. (San Francisco, CA). Animals were infused intravenously with 4 ml of plasma/kg body weight at an infusion rate of 1 ml/min.

### qPCR.

Quantitative real-time PCR assays were developed for detection of full-length genomic vRNA (gRNA), subgenomic vRNA (sgRNA), and total vRNA. Nasal lavages and tracheal aspirates were lysed in TRIzol LS, and RNA was extracted from the aqueous phase. RNeasy minikits (Qiagen) were used to purify the extracted RNA. Following DNase treatment (ezDNAse; Invitrogen), cDNA was generated using superscript IV reverse transcriptase (Thermo Fisher) in the presence of RNAseOUT (Invitrogen). A portion of this reaction was mixed with QuantiTect probe PCR master mix and optimized concentrations of gene-specific primers. All reactions were run on a QuantStudio 12K Flex real-time cycler (Applied Biosystems). gRNA was quantified by targeting orf1a-nsp4 using primers orf1a_F7 (GTGCTCATGGATGGCTCTATTA) and orf1a_R7 (CGTGCCTACAGTACTCAGAATC) with probe orf1a_P7 (/56-FAM/ACCTACCTT/ZEN/GAAGGTTCTGTTAGAGTGGT/3IABkFQ/). sgRNA was quantified using primers sgLeadSARSCoV2_F (CGATCTCTTGTAGATCTGTTCTC) and wtN_R4 (GGTGAACCAAGACGCAGTAT) with probe wtN_P4 (/56-FAM/TAACCAGAA/ZEN/TGGAGAACGCAGTGGG/3IABkFQ/). Total vRNA was quantified using primers wtN_F4 (GTTTGGTGGACCCTCAGATT) and wtN_R4 with probe wtN_P4. Standard curves generated from PCR amplicons of the qPCR targets were used to establish line equations to determine RNA copies/ml of sample.

### Clinical assessment.

Clinical monitoring was performed by a veterinarian each day, and sedated assessments were performed by the same veterinarian. Animals were sedated with ketamine-HCl (10 mg/kg i.m.) for the clinical assessment. Dexmedetomidine (15 μg/kg i.m.) was administered after clinical assessments to facilitate sampling, and midazolam (0.25 to 0.5 mg/kg i.m.) was added as needed. Radiographs were obtained with a HF100+ Ultralight imaging unit (MinXRay, Northbrook, IL) at 50 kVp, 40 mA, and 0.1 s. Blood pressure was obtained via oscillometry with a Vet25 and an appropriately sized cuff according to the manufacturer’s instructions (SunTech, Morrisville, NC). Oxygen saturation was obtained by pulse oximetry with a Radical 7 (Masimo, Irvine, CA). Radiographs were scored by a veterinary radiologist with experimental group and time point masked. Scoring was performed on a scale of 0 to 3 for each lung lobe or sublobe (18).

Of the 4 animals that received human plasma, 3 developed signs of a reaction, including vomiting. Both animals receiving normal plasma and one of the animals receiving convalescent plasma developed these reactions. Reactions were controlled with diphenhydramine (4 mg/kg i.m.) and ondansetron (0.2 mg/kg i.m.). On clinical assessment, there was no evidence of aspiration, and oxygen saturation remained good.

### Luminex assays.

Binding antibodies in serum were detected using four individual enzyme immunoassays and a multiplex microbead immunoassay. Nucleocapsid His tag recombinant protein expressed in insect cells (Sino Biological, Wayne, PA), BetaCoV/Wuhan/IVDC‐HB‐05/2019 spike protein trimers (amino acids 1 to 1208; GIAID number EPI_ISL_402121) with 6×His tags at the C terminus (Immune Technology Corp., New York, NY), spike protein receptor binding domain His tag recombinant protein expressed in HEK cells (GenScript, Piscataway, NJ), and heat-inactivated viral lysate (isolate USA-WA1/2020 propagated in Vero E6 cells [BEI Resources, Manassas, VA]) were each individually validated by enzyme immunoassays and used for CNPRC colony surveillance before being used to test these study samples (43). The antigens were then combined with internal positive (IgG, anti-IgG) and negative (uninfected cell debris) controls as a multiplex microbead panel on the Luminex platform (Luminex Corp., Austin, TX). Luminex assays have been shown to be a sensitive and specific platform for simultaneous detection of SARS-CoV-2 antibodies (23, 44). Briefly, the antigens were conjugated to carboxylated magnetic beads (Luminex, Austin, TX) that were mixed together in wells of a 96-well microtiter plate and incubated with test sera or controls diluted in phosphate-buffered saline (PBS)-0.05% Tween 20 with 2% Prionex blocking agent to capture specific antibodies. After washing with PBS-0.05% Tween 20, the captured antibodies were then detected by subsequent incubation with R-phycoerythrin (R-PE)-conjugated goat anti-human IgG previously shown to detect macaque IgG (Jackson ImmunoResearch, West Grove, PA). Following an additional wash, the wells were interrogated using a Luminex 200 array instrument. The detection system in the array reader uses two lasers to analyze the microspheres in a flow stream. The first laser identifies each microsphere and its associated antigen based on the fluorescent signature of the microsphere, and the second measures the amount of bound antibody (median fluorescent intensity [MFI]) using the fluorescent reporter molecule attached to the complex.

A nonhuman primate-specific quantitative multiplex microbead panel (R&D Systems, Minneapolis, MN) was used to detect and quantify cytokines on the Luminex xMAP platform (Luminex, Austin, TX). Sets of unique internally color-coded polystyrene microbeads coated with specific antibodies for each analyte were incubated with the samples to capture the specific analytes. After washing, the beads were further reacted with biotinylated detector antibodies followed by streptavidin-PE to label the immune complexes on the beads, forming a uniquely distinguishable four-member solid-phase sandwich for each analyte. After a final washing to remove all unbound material, the beads for each sample, control, and standard curve point were interrogated in a Luminex 200 dual laser instrument that monitors the spectral properties of the beads and amount of associated phycoerythrin (PE) fluorescence for each sample. xPONENT software was used to calculate the median fluorescent index and the concentration for each cytokine in each sample.

### Ortho VITROS S1 total Ig assay.

Serum samples were tested to measure anti-SARS-Cov2 total Ig level on the Ortho VITROS 3600 (VITROS CoV2T) at Vitalant Research Institute San Francisco (VRI-SF) and Creative Testing Solutions (CTS), following the manufacturer’s instructions. This test allows identification of total antibodies (IgG, IgM, IgA) against the SARS-CoV-2 virus S1 spike protein and was released under EUA (the first SARS-CoV-2 serology assay to receive EUA). Briefly, in this chemiluminescence assay, antibodies present in the serum are bound in a double sandwich configuration to the SARS-CoV-2 S1 spike protein antigen on the testing wells and to horseradish peroxidase (HRP)-labeled recombinant SARS-CoV-2 antigen in the liquid phase. HRP catalyzes the light signal that is captured by the instrument’s luminometer and classified as negative or positive based on a threshold signal/cutoff (S/C) of 1.0. Based on the FDA EUA instructions for use (IFU), the assay has 100% specificity based on testing normal donor samples and 100% sensitivity for samples collected >8 days after COVID-19 symptom onset. Based on distribution of S/C values, the assay has a wide dynamic range with reactive results ranging from 1 to 1,000, and testing can be performed with high throughput on multiple VITROS platforms.

### Serum biochemistry.

Biochemistry analysis on serum samples was performed using Piccolo BioChemistry Plus disks that were run on the Piccolo Xpress Chemistry Analyzer (Abbott), according to the manufacturer’s instructions. This panel includes alanine aminotransferase (ALT), albumin, alkaline phosphatase (ALP), amylase, aspartate aminotransferase (AST), C-reactive protein, calcium, creatinine, gamma glutamyltransferase (GGT), glucose, total protein, blood urea nitrogen (BUN), and uric acid.

### CD8 T-cell assay.

Peripheral blood mononuclear cells (PBMCs) were isolated at day 7 from whole blood collected in cell preparation tube (CPT) vacutainer tubes by density gradient centrifugation as described previously (45). Fresh PBMCs were stimulated with overlapping peptide pools representing SARS-CoV-2. All antigens were used at a final concentration of 2 μg/ml in a stimulation cocktail made using 0.2 μg of CD28 and 0.2 μg CD49d costimulatory antibodies per test. Unstimulated controls were treated with volume-controlled dimethyl sulfoxide (DMSO), and phorbol myristate acetate (PMA)/ionomycin-treated cells served as positive controls. Tubes were incubated at 5% CO_2_ at 37°C, and protein transport inhibitors, brefeldin A and monensin, were added 1 h later. Following a total of 6 h of stimulation, cells were immediately stained and fixed, and images were acquired. Fluorescence was measured using a BD Biosciences FACSymphony with FACSDiva version 8.0.1 software. Compensation, gating, and analysis were performed using FlowJo (version 10).

### Pseudovirus neutralization assay.

Fifty percent neutralization titers were measured using a pseudovirus assay as previously described (46).

### Genome sequencing.

RNA was subjected to amplicon and metagenomic sequencing. A fixed volume (3 μl) from each sample was used as input for SARS-CoV-2 amplicon sequencing (modified from reference 47 for Tn5-based Illumina adapter incorporation). For metagenomic sequencing, 10 to 100 ng of total RNA from each sample, together with a fixed mass (25 pg) of the external RNA control consortium RNA spike-in mix (ERCCS RNA spike-in mix, Thermo Fisher), served as input for metatranscriptomic next-generation sequencing (mNGS) library preparation (NEBNext Ultra II RNAseq library prep, New England Biolabs), as per the manufacturer’s instructions, with the following modifications: initial fragmentation time was reduced from 15 min to 12 min, and an incubation step with 1:10 dilution of FastSelect (Qiagen) reagent was included between the RNA fragmentation and first-strand synthesis steps of the library preparation to deplete highly abundant host rRNA sequences present in each sample. Equal volumes of individual SARS-CoV-2 amplicon library preps were pooled, and equimolar pools of individual mNGS library preps were pooled. Both pools were subjected to paired-end 15-bp sequence analysis on an Illumina NovaSeq 6000 to yield approximately 1 million reads per sample for the SARS-CoV-2 amplicon library preps and 50 million reads per sample for the mNGS library preps. Reads were aligned to the reference genome (GenBank accession MN908947.3) with minimap2 (48). ivar (49) was used to trim ARTIC primers, and bcftools was used to generate a pileup. We ascertained intrahost single-nucleotide variants (iSNVs) with ≥5% ALT allele frequency in at least one sample, requiring a coverage depth of at least 200 reads in that sample, following a rule of thumb requiring at least 1/*x* reads to ascertain an iSNV at frequency *x* (50). We quantified the degree of polymorphism within each sample using the nucleotide diversity at the ascertained sites. We validated iSNV allele frequencies by checking their concordance between amplicon and metagenomic sequencing for the nine samples that recovered over two-thirds of the genome in both approaches.

### Velox CoVAM assay.

Antibody responses to SARS-CoV-2, SARS-CoV, MERS-CoV, seasonal coronaviruses, and other various common cold viruses were assessed by Velox Biosystems Inc. using a modular microarray imaging assay as previously described (51).

### Statistics.

Statistical analyses were performed using GraphPad Prism version 9.0.2 for Mac OS X, GraphPad Software, San Diego, CA, USA (www.graphpad.com).
