# Supplementary material for: SARS-CoV-2 Infection of Rhesus Macaques Treated Early with Human COVID-19 Convalescent Plasma
Source: Microbiol Spectr. 2021 Nov 24;9(3):e01397-21. doi: 10.1128/Spectrum.01397-21 (PMC8612156; doi:10.1128/Spectrum.01397-21)

Fig. S1. Impact of CP treatment on viral burden. **(A, B)** Median vRNA and full-length (gRNA) levels in nasal lavages (A) and tracheal aspirates (B). The dashed line indicates limit of detection. **(C)** Results of Area under the curve analysis of total vRNA, full-length (gRNA) and sgRNA levels in nasal lavages and tracheal aspirates. Bars show mean with SEM. *n*=2 (CP treatment), *n*=2 (NP treatment), and *n*=4 (not treated).

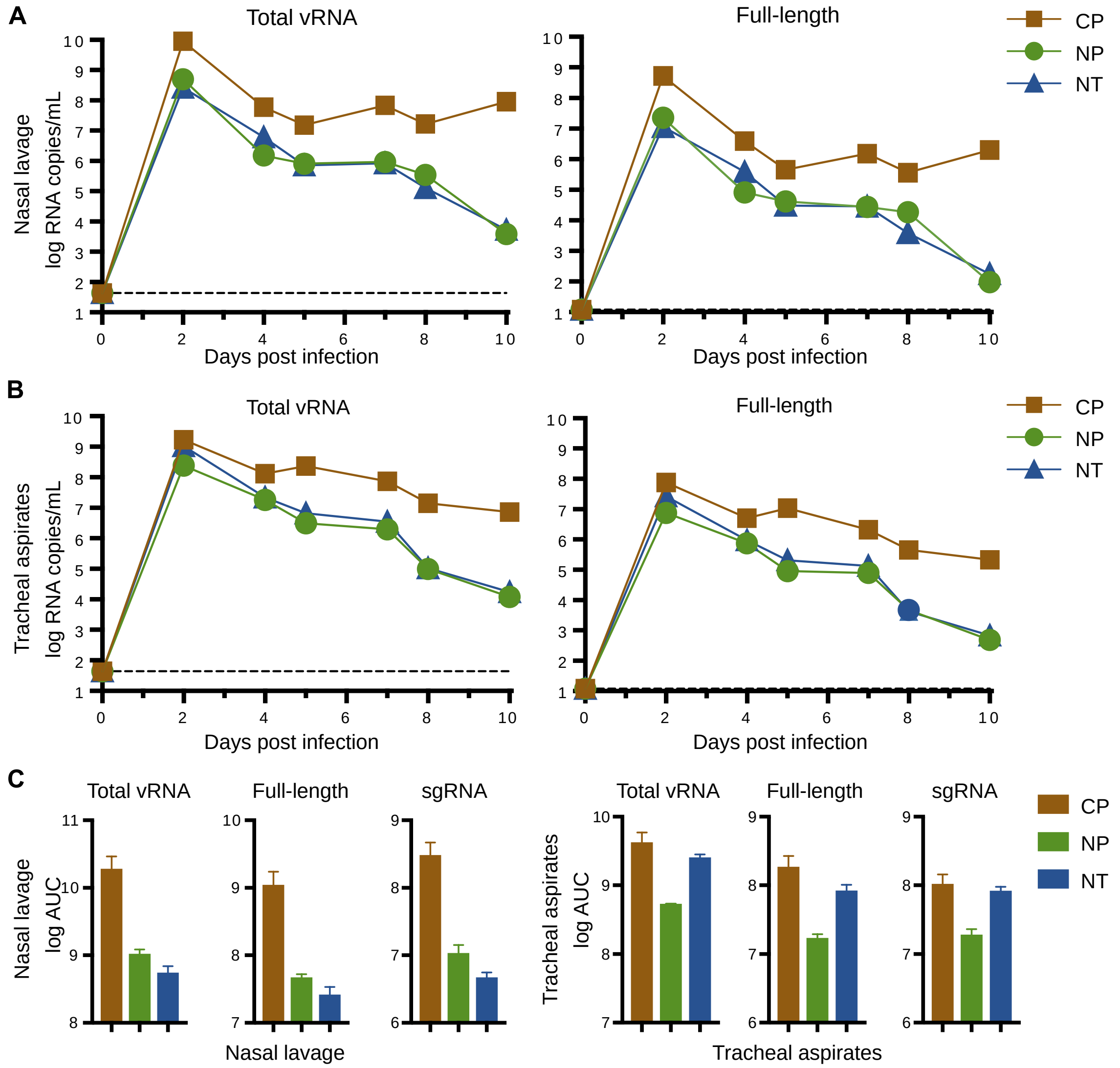

Fig. S2. Clinical markers of infection and COVID-19. Graphs show measurements in individual animals over time.

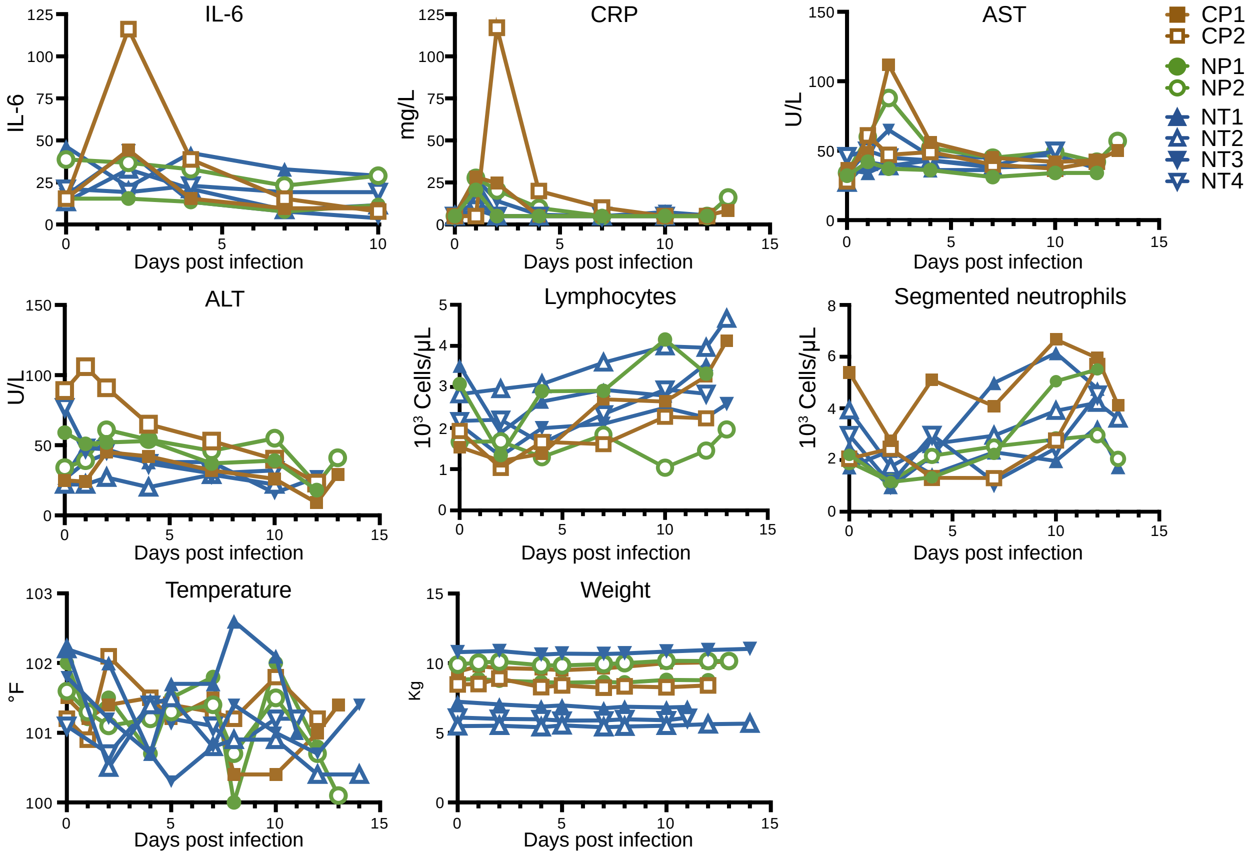

Fig. S3. Analysis of intra-host polymorphisms. **(A)** Read depths between the amplicon sequence approach (ARTIC) and metagenomic sequencing (mNGS). **(B)** Relationship between nucleotide diversity and viral loads. **(C)** Allele frequencies in recovered viral genomes.

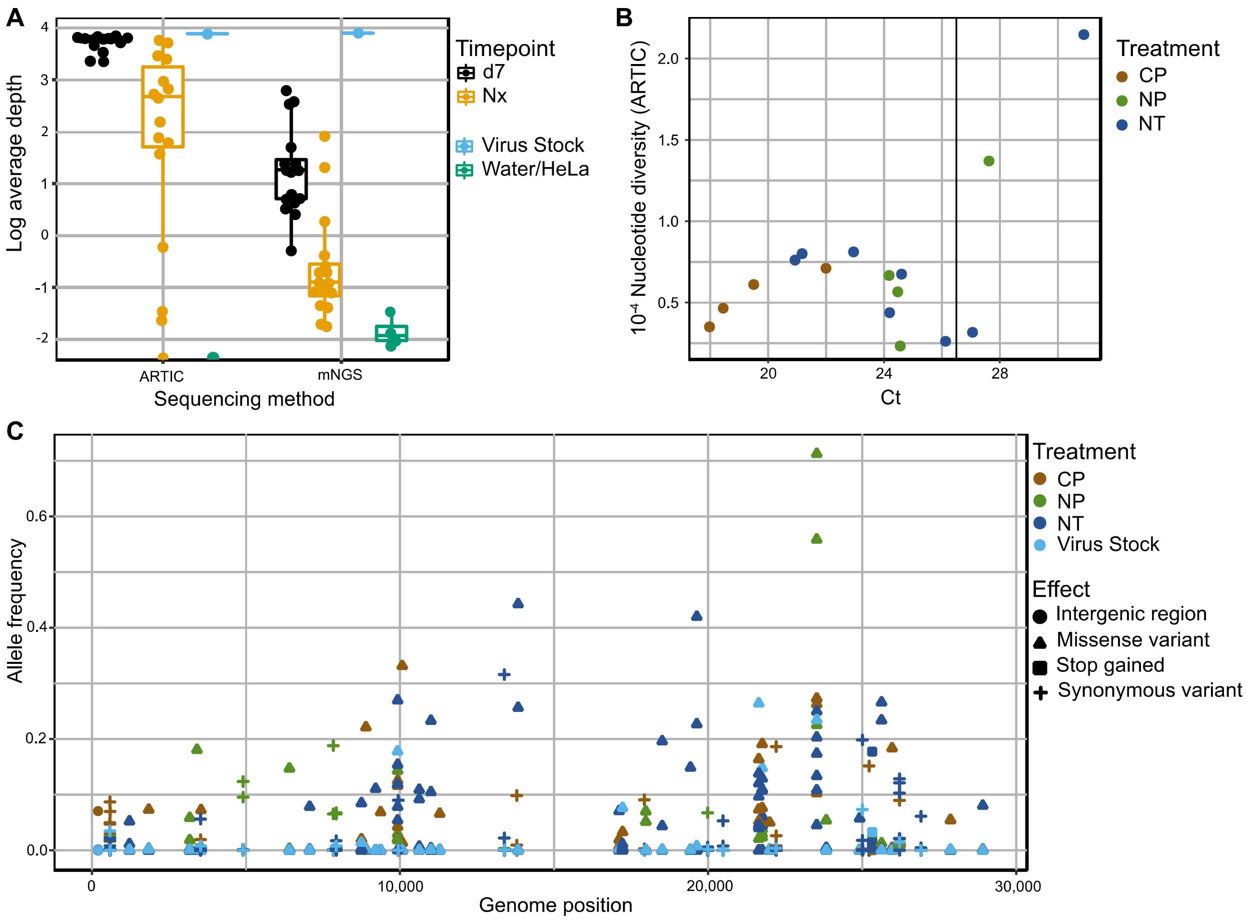

Supplement: SUPPLEMENTAL FILE 1 — Supplemental material. Download Spectrum.01397-21-s0001.pdf, PDF file, 0.2 MB [file spectrum.01397-21-s0001.pdf]
